# When primary care providers and smokers meet: a systematic review and metasynthesis

**DOI:** 10.1038/s41533-021-00245-9

**Published:** 2021-06-01

**Authors:** Emilie Manolios, Jordan Sibeoni, Maria Teixeira, Anne Révah-Levy, Laurence Verneuil, Ljiljana Jovic

**Affiliations:** 1 ECSTRRA Team, UMR-1153, Inserm, Université de Paris, Paris, France; 2 Service de Psychologie et Psychiatrie de Liaison et d’Urgences, Hôpital Européen Georges Pompidou AP-HP, Hôpitaux Universitaires Paris Ouest, Paris, France; 3 Service Universitaire de Psychiatrie de l’Adolescent. Argenteuil Hospital Centre, Argenteuil, France; 4 Université de Paris, ECEVE UMR 1123, Inserm, Paris, France

**Keywords:** Lifestyle modification, Health occupations

## Abstract

Primary Care Providers (PCPs) often deal with patients on daily clinical practice without knowing anything about their smoking status and willingness to quit. The aim of this metasynthesis is to explore the PCPs and patients who are smokers perspectives regarding the issue of smoking cessation within primary care settings. It relies on the model of meta-ethnography and follows thematic synthesis procedures. Twenty-two studies are included, reporting on the view of 580 participants. Three main themes emerge: (i) What lacks, (ii) Some expectations but no request, and (iii) How to address the issue and induce patients’ motivation. Our results reveal a global feeling of a lack of legitimacy among PCPs when it comes to addressing the issue of tobacco and smoking cessation with their patients, even though they have developed creative strategies based on what is at the core of their practice, that is proximity, continuity, long-term and trustworthy relationship.

## Introduction

Tobacco use kills up to half of those who use it (more than 8 million people a year), and there may be 1.1 billion smokers across the planet. Addiction to smoked tobacco depends on nicotine and involves an interplay of many factors (pharmacological, genetics, social, environmental, psychological, behavioral) resulting in an uncontrollable need to smoke so to modulate mood and arousal and relieve withdrawal symptoms. Over the past 20 years, the total number of people using tobacco worldwide has begun to fall for the first time, by around 60 million (from 1.397 billion in 2000 to 1.337 billion in 2018)^1^. This reduction is the result of comprehensive measures and actions undertaken at the national and international levels.

Smoking cessation intervention programs—*such as the 5A approach—Ask, Advise, Assess, Assist, and Arrange*^2,3^, *the motivational interviews*^4,5^ and *brief advice*—have shown efficacy, but among specific populations or in specialized clinical settings^6,7^. Professional support and cessation interventions or medications increase significantly the chance of successful quitting, while without support 95% of attempts to quit will fail^1^.

The World Health Organization (WHO) argues that primary health care is the most suitable health setting for providing advice and support on smoking cessation^8^, as it provides frequent and important opportunities to identify tobacco use, provide advice and help people to quit^9,10^. Yet, despite being an opportunistic and trustworthy setting^11,12^, many smokers do not receive support from their primary care providers (PCPs)^13^, since only a few of them have received training in delivering specific interventions^14^, and most of the patients come in daily clinical practice without an explicit demand of quitting.

Most of the national guidelines focus either only on “smokers who want to stop”^15^, or are based on the 5A-approach supposedly covering every stage of the process^16,17^. There are many guidelines for smoking cessation in primary care—clinical practice guidelines, national recommendations, public health policies; they all address the need to identify smokers, to deliver behavior change intervention, to advise patients to quit, and offer cessation interventions or medications^17–20^. However, some inconstancies and gaps within the guidelines need to be underlined: (i) these recommendations do not detail how PCPs should do to achieve these goals, (ii) only a few of them, among guidelines from 22 countries, have involved PCPs directly in their development^21^, (iii) only two guidelines have included recommendations for “a person who smokes [and] is not ready to quit”^22,23^, based on prevention campaigns main lines—understanding the risk and encouragement to seek help to stop—and taking into account the patient’s own time frame and personal needs and goals^22^.

Quantitative literature focuses mostly on the smoking cessation phase^24–28^, the obstacles professionals encounter in initiating smoking cessation treatment^29,30^, and the question of prevention^31–33^. Conducting qualitative research is becoming essential in the field of addiction in general, tobacco addiction in particular, in order to better inform policies by more patient and public involvement. Qualitative studies are relevant to explore complex issues such as tobacco use and to find new ways to improve smoking cessation outcomes in primary settings through in-depth descriptions of the lived experience of PCPs and patients in great depth. Because qualitative studies are usually conducted with small samples and in specific contexts, there may often be concerns about the generalizability of their results. Synthesizing data from qualitative studies can help in the development of health policies and clinical practices. Yet, to date, no systematic review of this qualitative literature has ever been conducted.

We thus conducted a systematic review and metasynthesis of qualitative studies^34,35^ in order to explore the lived experience of both PCPs and smokers regarding tobacco use and smoking cessation when meeting in this specific setting.

## Results

**Fig. 1:**
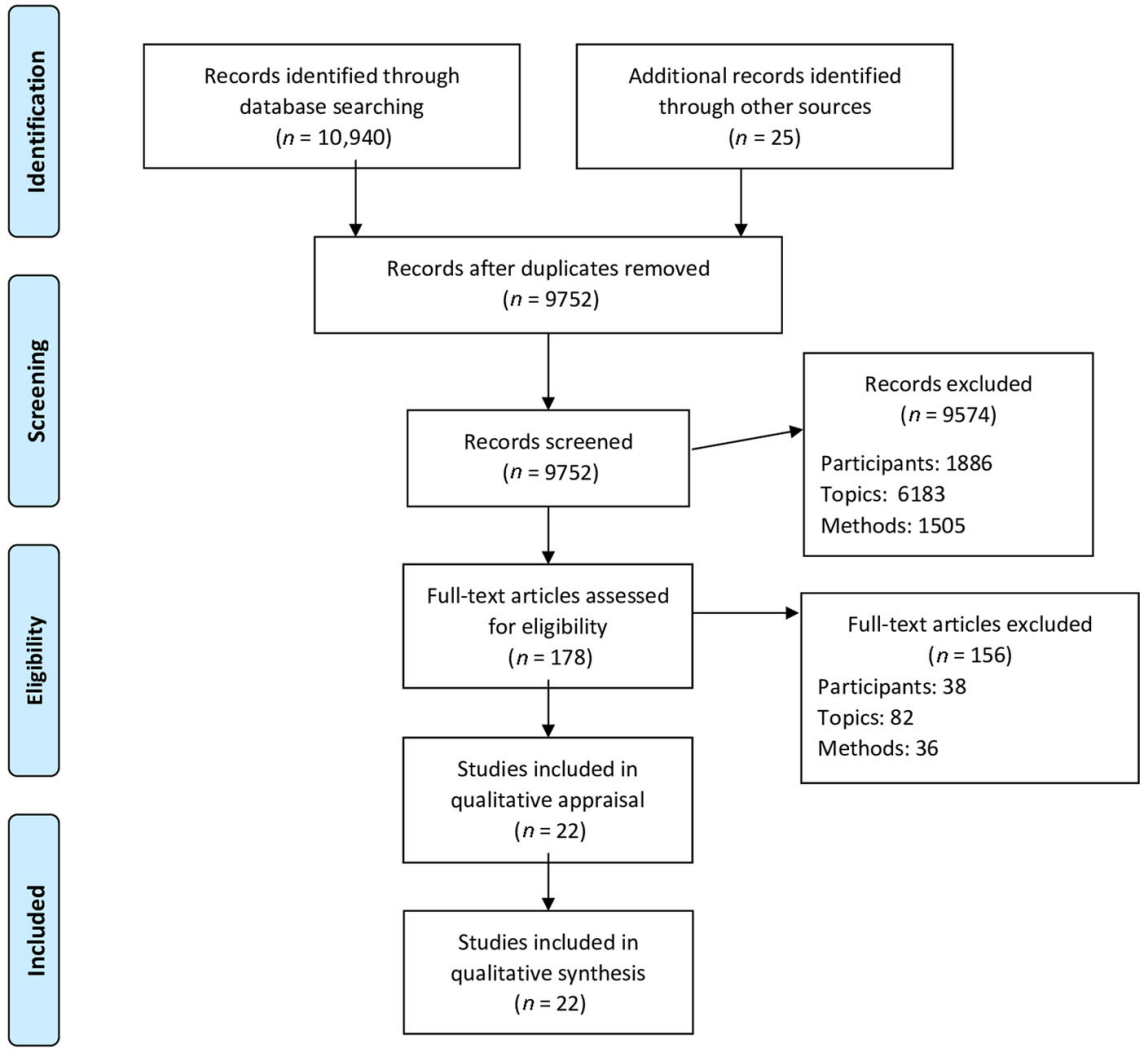
Flow of information through the different phases of the study selection. From: Moher, D., Liberati, A., Tetzlaff, J. & Altman, D. G. The PRISMA Group. Preferred Reporting Items for Systematic Reviews and Meta-Analyses: The PRISMA Statement. *PLoS Med.* 6(6), e1000097 (2009).

Of the 10940 articles initially retrieved, 22 studies were included (Fig. 1). Participants were patients (*N* = 325) (current smokers *N* = 289, ex-smokers *N* = 36) and primary care providers (*N* = 255) (nurses *N* = 50; including 31 smokers), general practitioners (*N* = 159), residents (*N* = 14), dentists (*N* = 23)).

These studies came from six English-speaking countries. Table 1 describes the global characteristics of the included studies (Supplementary Table 1 describes the characteristics of each study).

**Table 1:** Summary of the main characteristics of the included studies.

Years of publication	2000 (*N* = 1)	2010 (*N* = 3)	2016 (*N* = 1)
	2003 (*N* = 1)	2011 (*N* = 1)	2018 (*N* = 1)
	2004 (*N* = 2)	2012 (*N* = 2)	2019 (*N* = 1)
	2007 (*N* = 1)	2013 (*N* = 1)	2020 (*N* = 1)
	2008 (*N* = 1)	2014 (*N* = 1)	
Countries	Europe	America	
	United-Kingdom (*N* = 8)	United States (*N* = 4)	
	Denmark (*N* = 3)	Canada (*N* = 1)	
	Sweden (*N* = 1)	Asia	
	Poland (*N* = 1)	Malaysia (*N* = 1)	
	Spain (*N* = 1)	Australia(*N* = 1)	
	The Netherlands (*N* = 1)		
Settings	General population (*N* = 3)		
	Primary care settings (*N* = 6)		
	General practice consultation (*N* = 7)		
	Primary care clinics (*N* = 2)		
	Dental care (*N* = 2)		
	Other (*N* = 2)		
Participants	patients (*N* = 325)		
	Current smokers *N* = 289		
	Ex-smokers *N* = 36		
	primary care providers (*N* = 255)		
	Nurses (*N* = 50 including 31 smokers)		
	General practitioners (*N* = 159)		
	Residents (*N* = 14)		
	Dentists (*N* = 23)		
Data collection	Semi-structured interviews (N = 17)		
	Focus Group (*N* = 4)		
	Video recording (*N* = 1)		
Data analysis method	Content analysis (*N* = 9)		
	Thematic analysis (*N* = 6)		
	Giorgi’s four-step process (*N* = 3)		
	Straussian grounded theory method (*N* = 1)		
	Phenomenological approach (*N* = 1)		
	Conversation analytic principles (*N* = 1)		
	Framework analysis approach (*N* = 1)		

The quality appraisal showed that the overall quality of the studies was high (Table 2, Supplementary Table 2). Several papers failed to address the role of the researchers’ contribution to the findings and/or interpretations (reflexivity item, 21 studies). The CERQual assessment of the findings showed “high confidence” or “moderate confidence” in most of the categories (Supplementary Table 3).

**Table 2:** Summary of quality appraisal.

Principal criterion	Specific criteria specific (non-exhaustive list)	Quality assessment of the studies		
		Yes	Partially	No
Objectives	Explicit, relevant, important objectives	22	0	0
Method	Appropriate use of qualitative methods	22	0	0
Design	Design justified by the authors	21	1	0
Recruitment of participants	Recruitment described, appropriate, and justified by the authors	21	1	0
Data collection	Mode of collection clear, adequate, justified by the authors, data saturation discussed	21	1	0
Reflexivity of researchers	Researchers reflected on their own role and potential biases at different stages of the study	1	0	21
Ethical considerations	Approved by an ethics committee, details to participants	22	0	0
Data analysis	A specific description of the data analysis process, data sufficient to support the results	22	0	0
Results	Explicit, credible, discussed results	21	1	0
Value of the study	Contribution to existing knowledge, transferability, identification of new avenues of research	22	0	0

Three central themes emerged from the analysis: (i) What lacks (ii) Some expectations but no request and (iii) How to address the issue and induce patients’ motivation. Supplementary Table 4 presents excerpts of transcripts quoted in the articles studied, selected to exemplify the results described.

### What lacks

Patients, PCPs, and authors enumerated many things that lacked in order to successfully lead patients to an active or specialized smoking cessation intervention.

#### Patients’ lack of motivation

Patients described a lack of internal motivation to stop smoking and stated that their own will and motivations were the key to quit^36^. Many were ambivalent about quitting smoking or not, rationalizing between some negative aspects (health, cost…) and positive ones (pleasure, habit…)^37^.

#### PCPs’ lack of sincerity and adequacy

*S*ome professionals felt that they were not in a position to raise the subject because they smoked themselves. They described a feeling of hypocrisy and felt uncomfortable and inadequate^38,39^. Many nurses who were smokers denied participating directly in habit-breaking therapies since they felt uncomfortable helping others to break a habit they could not control themselves^38^.

#### Lack of support

The authors reported a lack of institutional support^40^. Some PCPs described a feeling of solitude because they had to handle these issues completely on their own^40^.

#### Lack of time and of a common time frame

Because of their workload, PCPs lacked time to enter into a time-consuming process of initiating and providing support to their patients with tobacco addiction from a “no-request” position to smoking cessation interventions^41–43^.

#### Lack of skills and training

Many PCPs considered their skills in this area to be mediocre and their knowledge inadequate; they also described a feeling of poor self-efficacy^40,44,45^.

### Some expectations but no request

In these studies, patients described having different kinds of expectations related to their tobacco use when meeting PCPs, even if they did not disclose any explicit request. Accordingly, PCPs did not act the same in front of this absence of a request.

#### Patients’ expectations about PCPs

Some patients expected nothing from their PCPs, that is, they did not want any advice or even the subject to be raised in consultations^39,41,46^. They argued that their tobacco use problem was their problem only and their own responsibility^43^ and that quitting was only a matter of their own will to start the process^47^. Similarly, other patients stated that the “smoking cessation topic” was to be initiated by themselves only and not by PCPs, except when it was directly relevant to the medical issue they were seeking help for. Some PCPs in these studies shared this view^44^. However, other patients, in line with most of the PCPs in these studies, considered that PCPs were doing their “duty” when systematically exploring smoking status and habits^43^. According to some patients, tobacco use needed to be a topic regularly and routinely discussed during consultations^41^. Patients expected from PCPs to show support and motivation the moment they would ask for help^41^. That echoes a commune PCPs attitude in these studies, not acting before a request but be proactive and supportive as soon as a patient explicitly stated that he/she wanted to quit smoking^43^.

Patients expected PCPs to respect their own rhythm and timing^48^. According to some authors, both patients and PCPs valued a non-moralist and non-judgmental approach, so patients could feel free to speak about everything^48^.

Finally, many patients expected an active role from PCPs^49,50^: to give advice to every patient about smoking^46–48,51^ and to provide education, information, and smoking cessation options^41,49^. Some patients explained that they were too ashamed to admit they were smoking and would not be able to raise the issue by themselves^49^. According to the patients, general practitioners (GPs) were at the best place to initiate the process—better than specialized clinics-, since they knew the patients the best and had an established ongoing relationship with them^41,46,51,52^.

#### PCPs roles and attitude

All the PCPs included in the studies were fully aware that they had a role to play in their patients’ smoking cessation process. Yet, they did not agree on which role and the level of involvement and responsibility they needed to have.

Some PCPs limited themselves to the following objective: to plant a seed for the long term^40,53^. GPs considered their role was to provide advice and referral to the nurses for assistance, but not to motivate every smoker to stop smoking^43^. They felt the burden was out of their hands after advising^43^.

As already mentioned, a common attitude among PCPs was to wait for an explicit request. PCPs wanted to preserve the therapeutic alliance and addressed smoking issues only if patients initiated the talk first^41,54^. These PCPs met the patients’ expectations to respect their rhythm and timing and to offer professional support as soon as the patients requested it^48^.

Finally, many PCPs in general, and GPs in particular, felt they had a responsibility toward their patients who used tobacco. The question was not *if* it was their role to support smoking cessation, but *how* they should do it^42^: investigating patients’ smoking status, as part of a routine intake or annual physical^41^, providing advice and options^43^, educating people about the smoking health outcomes^41^, but also inducing motivation to quit.

### How to address the issue and induce patients’ motivation

#### Timing and temporality: the right moment

Both patients and PCPs underlined the impact of finding the right moment to speak about smoking cessation, that is either when the patient was ready to speak about it^48,55^ or when the context of the consultation made it a relevant issue to address, for instance when patients had a disorder potentially aggravated by smoking, such as asthma, cardiovascular diseases, diabetes, or periodontal problems^41,42,51,56^. The authors described the concept of a ‘teachable moment’^56^.

#### Familiarity, continuity, and trust

What appeared predominantly was the importance of the relational aspects among patients and PCP. Many patients underlined the unique relation of trust they had with their GPs^51,52^. A long-term relationship, based on long acquaintance and familiarity^42^, and a genuine dialog between professionals and patients were seen as key factors and therapeutic levers to enhance smoking cessation^40^. Authors mentioned “ongoing, longitudinal relationships with patients”^57^, building trust over time^40,46^, as an efficient way to reinforce success or to suggest other solutions to overcome the failures and to take the advice given acceptable and “hearable“^46^. Therapeutic relationship was the most salient result for several authors^40,49^. Some nurses who were smokers even thought that their tobacco use could facilitate the therapeutic relationship^38^.

#### Strategies and approaches

Both patients and PCPs have experienced many strategies and approaches.

#### Testing the water, small talk, or holistic method

For patients, good practices involved using a respectful tone, sensitivity to the patient’s receptivity, understanding the patient as an individual, being supportive, and neither “preaching”^47^, nor “pushing”^41^. On that matter, some PCPs used a “testing the water” strategy, that is to introduce the subject in “baby steps“^58^—using verbal and non-verbal cues to assess whether patients were motivated to stop smoking or not^57^—or “small talk”^42^ taking every opportunity to provide the information, especially with humor^40^. Others described raising the question of smoking as part of a holistic approach to medicine^42,54^.

#### Rational strategy and tangible link

Motivation for quitting resulted from personal impact and tangible prompts, when patients could physically see the damage caused by smoking^56^. PCPs used a rational strategy linking patients’ symptoms to their smoking^42,58^ and thus legitimizing the fact they had to address this issue^49^.

#### A “collaborative strategy”

Both underlined the importance of PCPs having an approach, based on respect and comprehension^41,47,51^. PCPs considered this approach as part of a relationship of trust that supports, encourages, and sustains behavioral change, with an idea of mutuality in the conversation^40,46,48,58^. Moreover, both expressed the need for more direct and more frequent verbal discussions^48,51^. For example, PCPs could open a discussion by stating that they were aware that the patient had earlier said “no” and that they just wanted to know if the patient had changed his/her mind^48^.

#### A “confrontational strategy”

Few patients suggested that PCPs should try to scare them into quitting, with visual images illustrating the health consequences of smoking. Yet, paradoxically, the same patients stated they would not quit if confronted by a major personal smoking health shock^47^. This confrontation strategy was described by PCPs as “firm”, “strong”, “more direct”, “more forceful,” and “telling patients off,” but also criticized as nagging”^58^. Some underlined rigor and direct communication^40^, others “scare tactics” to highlight the harmful effects of smoking^44^.

#### Patient-centered approach

Patients valued the advice given in an individualized context, if not they would associate them with a public health campaign and would not feel personally concerned^41,47,49^. Patients without request felt neither listened to nor recognized when PCPs focused on their smoking^54^. PCPs also underlined a more tailored approach^44^.

#### Educational approach

Several patients pointed out that materials should be available for review before meeting with the PCP^51^. Many PCPs described educating their patients on the health risks of smoking^44^.

#### Using addiction model

Patients recognized smoking as a serious addiction^51^ and many PCPs used it as a way to address it, recognizing the addictiveness of nicotine and the difficulty of quitting^41^.

Patients finally suggested two strategies unfound among PCPs: *positive and targeted messages*^37,51^ and *carbon monoxide monitoring*^51^.

## Discussion

Our results underlined the many obstacles perceived by the PCPs and the patients, but also the creative strategies used by PCPs in their daily practice.

Many obstacles are already addressed in the literature: lack of clinician engagement, lack of clarity of the policies and guidelines, lack of time, lack of resources, lack of managerial support, lack of training, healthcare professionals’ negative beliefs^59–66^. PCPs workload and increasing number of patients impose them very often to reduce the time of their appointments^67^. Addiction medicine is not mandatory in GPs—and other PCPs—training and when integrated, only a little time in the curriculum is devoted to substance use disorders and addictions^68^. As for specific training regarding tobacco use addiction, many models, tools and theories have been developed and can be applied in PCPs daily practice, for instance, the 5As and 5Rs—to both assist smokers willing to quit (5As) and implement interventions designed to increase future attempts with patients unwilling to quit at the time of the visit (5Rs)^69^, or the transtheoretical model by Prochaska and DiClemente^70^, the label “teachable moment”^2,71^ and “opportunistic smoking cessation interventions”^72^, such as the “ flashcard for a motivational-based intervention” tool, taking only 30 s to 3 min to use^73^. Moreover, some national public health policies directly target and compel healthcare professionals to deliver opportunistic health behavior change interventions to patients during routine medical consultations, for instance, the “Making Every Contact Count”^74^ campaign in the United Kingdom^75^. Yet, a study has shown that only 31,4% PCPs had heard about this policy^76^. As a matter of act, rates of tobacco treatment delivery in primary care are quite low^77^, research has shown that PCPs had a suboptimal adherence to smoking cessation guidelines^78^, and that, even informed less than 50% of them, in only 50% of occasions, would offer adjunct support to patients^73,76,79^.

All of these obstacles and difficulties, plus the fact that some PCPs are smokers themselves, contribute to an overall feeling of a lack of legitimacy.

This is an original and unexpected point raised by our metasynthesis that would need further research. This encounter between PCPs feeling not legitimate and a smoker who has neither a request nor motivations to quit could explain why, opportunities created by a primary care setting are very often missed, despite all the specific guidelines, tools, and policies^73,80–82^. However, our results show that smokers do perceive PCPs as legitimate and at the right place to address their tobacco issue. This strengthens the positions of several quantitative studies^73,83^. Most of the patients expect help from PCPs regarding their tobacco addiction. Moreover, what makes PCPs legitimate to help their patients who are smokers is not related to any specific skills or knowledge in addiction, psychiatry, or psychotherapies, but to what is the core of PCPs practice, that is proximity, continuity, long-term and trustworthy relationships. Ideally, all PCPs should receive proper addiction medicine training, but their role is not to be an attenuated version of a tobacco specialist or a therapist. In other words, rather than focusing on addressing what lacks, we should focus on PCPs specific knowledge and skills. PCPs expertize and specific relation with the patients make the primary care setting a relevant opportunistic situation to initiate and support patients’ smoking cessation^84^. PCPs need to be aware of their important role and allow more time to this important task^72^. They need to overcome their feeling of illegitimacy and the absence of patients’ request^84^ and focus on how inducing a will to quit to a patient who came to visit them for another reason.

Our results suggest some practical implications for PCPs when meeting a patient who is a smoker:

- (i) being proactive without waiting for patients explicit request while taking into account their own time frame and personal needs and goals^22^;
- (ii) integrating the tobacco use as a regular and routine issue to discuss in the global assessment of the patient’s daily life^84^;
- (iii) investigating the relationship the patients are entertaining with its tobacco use not in a binary way (to want to quit or not, to be motivated or not) but with open questions—*what is it for you to be a smoker? what do you think of your tobacco use?*—so to explore pros and cons, doubts, worries, and expectations.

Yet caution will be required to transpose these practical implications in cultural contexts not represented in the studies included. Conducting implementation research and transcultural studies to ensure their local relevance would be necessary.

Further qualitative and quantitative research is necessary to in-depth explore the feeling of illegitimacy among PCPs and to better integrate PCPs specific skills and competencies in guidelines, so they can truly be in the frontline to effectively prevent tobacco addiction and its harmful effects.

This metasynthesis includes the experience of 325 participants. The method we applied is rigorous, has been tested in medical research^85^, and meets the criteria of the ENTREQ guidelines^86^.

Nonetheless, certain aspects of this metasynthesis limit the generalization of its conclusions. A qualitative metasynthesis collects only partial data from the participants and the interpretations of the researchers, which are the data given in the initial articles. Moreover, although the review assembled articles from diverse cultural areas, English-speaking countries are overrepresented as we restricted our selection to articles in that language. That’s why the conclusions of this study might be restricted to this cultural area.

Finally, the results of the studies included in this metasynthesis were redundant from a methodological perspective. More participatory research methods are needed to involve professionals and patients so to reach more original and relevant results.

## Methods

This meta-synthesis relies on the model of meta-ethnography^35^ and follows the thematic synthesis procedures described by Thomas and Harden^34^.

Inclusion criteria were as followed.

### Participants

We selected studies exploring tobacco issues among patients who smoke and PCPs (either doctors, nurses, dentists, etc…), i.e., the stakeholders involved in this encounter. In order to remain as close as possible to daily practice in primary care settings, we decided not to include PCPs with specific training—both theoretical (about tobacco or addiction) and practical (specific interventions) or any tobacco-related specialist (PCPs working in tobacco clinics, pneumonologist, addiction specialist).

### Outcomes

Participants reported experiences during primary care consultations about the issue of tobacco use and smoking cessation.

### Studies

Qualitative studies, based on a well-known qualitative methodology and using specific data collection tools and data analytical procedures (Table 3).

**Table 3:** Inclusion and exclusion criteria.

	Inclusion criteria	Exclusion criteria
Design	Qualitative research	Quantitative and mixed studies
Article type	Peer-reviewed journal article	Reviews, commentaries, editorials, thesis, non–peer-reviewed journal articles
Language	English	Other than English
Participants	Doctors, nurses, healthcare providers that are non-trained and non-specialists	Specialists, trained professionals
	Smokers	
Topic	Related to the smoking cessation in primary care	
Countries	All countries	

As for exclusion criteria, we excluded studies including healthcare providers who were specifically trained in smoking cessation interventions or working in tobacco-related settings. Studies that focused on the perinatal period were not included because that would require a metasynthesis on its own (Table 3).

Search Strategy involved screening from four databases: MEDLINE, PsycInfo, CINAHL, and SSCI from April through November 30, 2019, with an update in October 2020 (Supplementary Table 5). Preliminary research had identified several articles from which we selected keywords. Extensive lateral searches also sought to identify papers that might have eluded our algorithms.

Study selection was done after collecting the references and eliminating duplicates, two authors (J.S. and E.M.) subsequently read the titles and abstracts to assess their relevance. The potentially relevant articles were then read in full, and a second selection made to keep only the articles that met our inclusion criteria. Disagreements were resolved during meetings of the research group.

Two authors (E.M., J.S.) assessed the quality of included articles independently by applying the Critical Appraisal Skills Program (CASP) criteria to each (Table 3; Supplementary Table 2).

Then they discussed the results within the research group until an agreement was reached. No study was excluded from the analysis based on this evaluation.

One researcher (E.M.) extracted the formal characteristics of the studies and three (J.S., E.M., and A.R.L.) independently and exhaustively extracted the first-order results (that is, the study results) and the second-order results (the authors’ interpretations and discussions of the results) in the form of a summary for each study we included (Supplementary Fig. 1). We endeavored to preserve the context of the studies included by reporting the essential characteristics of each.

Qualitative data were analyzed thematically relied on an inductive and rigorous process. Three researchers (E.M., J.S., A.R.L.) independently and simultaneously conducted a descriptive analysis intended to convey the participants’ experience—from their own perspective and that of the authors. This involves, for each article and each researcher: (1) reading the summaries related to the article; (2) open coding each summary into—not predetermined—descriptive units; (3) categorizing the units, that is, regrouping them accordingly to their proximity of meaning and experience. These stages were carried out with the help of N’Vivo-12 software (QSR International). Progressively, each researcher conducted a cross-sectional analysis of all of the data analyzed thus far, by regrouping similar categories and excluding none of them. After this analysis, the three researchers met with the rest of the research group —who had read and become familiar with the data during this time—to share the categories uncovered. During these two-hour meetings, the group performed the work of translation, that is comparing and assembling the categories obtained by the analysis of each article to develop the key themes that captured similar ideas from the different articles and then to develop overarching concepts about the research question. In practice, the group had to: (1) regroup the categories into themes, a reorganization that uncovered the framework of the participants’ experience; (2) determine the key themes, that is, the most significant and relevant themes. Only four meetings were necessary to obtain the results as the level of agreement of those meetings was high. The high level of rigor of the results was obtained by triangulation of both the data sources and the analyses: three independent analyses and regular research meetings.

The CERQual (Confidence in the Evidence from Reviews of Qualitative research) GRADE approach^87^ was used to assess confidence in the findings of the metasynthesis, following four key components: methodological limitations, relevance, coherence, and adequacy of the data (Supplementary Table 3).

Assessment of these four components enabled us to reach a judgment. about the overall confidence for each review findings, that is, each category in our results, rated as high, moderate, low, and very low, with “high confidence” being the starting assumption^88^.

## Supplementary information

Supplementary Information

## Data Availability

All relevant data are available upon relevant and reasonable request. Researchers who are interested can write to the corresponding author of this publication jordansib@hotmail.com.
