# Supplementary material for: When primary care providers and smokers meet: a systematic review and metasynthesis
Source: NPJ Prim Care Respir Med. 2021 Jun 1;31:31. doi: 10.1038/s41533-021-00245-9 (PMC8169673; doi:10.1038/s41533-021-00245-9)
Supplement: Supplementary file 1 — Supplementary Information [file 41533_2021_245_MOESM1_ESM.pdf]

Supplementary information file

Supplementary table 1. Main Characteristics of the included studies

Authors	Year	Objective(s)	Country	Setting	Participants	Data collection	Method of analysis
Andersson et al.	2012	To elucidate dental hygienists' experiences of working with patients who smoke or use snuff toward smoking cessation	Sweden	The dental hygienists' workplace or home	Dental hygienists: 12	Semi-structured interview (SSI)	Content analysis
Bartlett et al.	2016	To report the experiences of smokers who are not ready to quit and explores the types of intervention approaches that might engage them.	United Kingdom	General population	Smokers:32	5 FG	Thematic analysis
Bell et al.	2011	To explore smokers' and GPs' perspectives on smoking cessation interventions in primary care settings.	Canada	Primary care settings	Smokers: 25<br>GPs: 10	SSI	Content analysis
Buczkowski et al.	2013	To determine what kind of general practitioner smokers need in order to stop smoking.	Poland	General population	Smokers: 24	4 FG	Content analysis
Butler et al.	1998	To determine the effectiveness and acceptability of general practitioners'opportunistic antismoking interventions by examining detailed accounts of smokers' experiences of these.	United Kingdom (Wales)	21 Primary care settings	Smokers: 32 and recently quit: 10	SSI	Thematic analysis
Champassak et al.	2014	To explore doctors' perceptions of useful strategies to motivate patients to quit, how receptive they felt patients were to these strategies, and the benefits and drawbacks of discussing smoking cessation with patients.	United States	Primary care outpatient clinic	Doctors and resident doctors: 14	SSI	Content analysis
Chean et al.	2019	To construct a model of the barriers to smoking cessation in the primary care setting.	Malaysia	Primary care setting (from previous study)	Smokers with at least 1 failed quit attempt: 57	SSI	Straussian grounded theory method
Coleman, Murphy & Cheater	2000	To elicit, relate, and interpret GPs' accounts of why they discuss smoking with some patients and not others.	United Kingdom	General practice consultation	GPs: 39	Video-recorded and SSI	Thematic analysis

Coleman, Cheater & Murphy	2004	To investigate how GPs believe they should advise smokers to stop and the reasons underpinning these beliefs.	United Kingdom	General practice consultation	GPs: 27	SSI based on consultation	Thematic analysis
Gonzalez et al.	2009	To identify the perception of Primary Health Care (PHC) female nurses in the Balearic Islands in Spain who are smokers, regarding the suitability of their anti-smoking therapeutic relationships with their clients.	Spain	Primary care settings	Nurses who smoked: 15	SSI	Content analysis
Guassora and Baart	2010	To identify frames of interaction that allow smoking cessation advice in general practice consultations.	Denmark	General practice consultation	GPs : 6<br>Smokers: 11	Observation + SSI	Giorgi's four-step process
Guassora and Gannik	2010	To describe how the process of developing and maintaining trust is related to how and if smoking cessation advice is given in general practice consultations.	Denmark	General practice consultation	GPs: 6<br>Smokers: 11	SSI based on observations	Giorgi's four-step process
Guassora and Tulinius	2008	To describe consultations in Danish general practice as a context for a mass strategy of smoking cessation advice.	Denmark	General practice consultation	GPs: 6<br>Smokers:11	SSI	Giorgi's four-step process
Halladay et al.	2015	To explore smokers' personal interactions with health care providers to better understand what it is like to be a smoker in an increasingly smoke-free era and the resources needed to support quit attempts and to better define important patient-centered outcomes.	United States	3 Primary care clinics	Smokers: 33	3 FG	Content analysis
Heath et al.	2004	To explore how tobacco-dependent nurse practitioners (NPs) in Minnesota describe their experiences with health promotion and disease prevention practices with patients who smoke.	United States	Areas of practice included trauma, neurology, cardiology, and primary care	Nurses who smoked: 16	SSI	Phenomenological approach
Holliday et al.	2020	To explore the views of smokers with periodontitis receiving dentist-delivered SCA (smoking cessation advice).	United Kingdom	Dentist's workplace	Smokers:28	SSI	Thematic analysis

Kerr, Watson & Tolson	2007	To explore the knowledge, attitudes and practice of members of the primary care team in relation to smoking/smoking cessation in later life.	United Kingdom (Scotland)	Primary care team	Nurses=41<br>GPs: 16	SSI	Content analysis
Lin and Ward	2012	To understand why some people continue to smoke in spite of well-known adverse health effects, what and how resilience factors impact on people's smoking, and the role and limitations of the GP in fostering resilience to smoking.	Australia	General population	Smokers: 17	SSI	Content analysis
Nowlin et al.	2018	To assess the extent to which dental practices in northeastern North Carolina were implementing the recommended U.S. Public Health Service (PHS) Clinical Practice Guideline, to understand policies about smoking in dental practice settings, and to identify barriers to implementation of recommended systems.	United States	Workplace	Licensed, practicing general dentists: 11	SSI	Thematic analysis
Pilnick and Coleman	2003	To describe factors influencing the provision of advice by GPs to smokers, and to develop a method for describing smokers' responses	United Kingdom	General practice consultation	GPs: 39<br>Consultations: 47	Videos recording	Conversation analytic principles
Van Rossem et al.	2015	To explore barriers and solutions of smoking cessation treatment, from the perspective of smokers and healthcare professionals in Dutch primary care	The Netherlands	Primary care settings	Smokers: 14<br>GPs and Practice nurses: 9	FG	Content analysis
Wilson et al.	2010	To explore how smokers motivated to quit are managed in the GP consultation, specifically how treatment and referral are negotiated from the perspectives of both parties	United Kingdom	General practice consultation	GPs: 10<br>Smokers: 20	SSI	Framework analysis approach

Supplementary Table 2. Evaluation of study quality according to CASP[1]

References	Aims	Methods	Research design	Sampling	Data collection	Reflexivity	Ethical issues	Data analysis	Findings	Value of research
Andersson et al.	y	y	y	p	y	y	y	y	y	y
Bartlett et al.	y	y	y	y	y	n	y	y	y	y
Bell et al.	y	y	y	y	y	n	y	y	y	y
Buczkowski et al.	y	y	y	y	y	n	y	y	y	y
Butler et al.	y	y	p	y	y	n	y	y	y	y
Champassak et al.	y	y	y	y	y	n	y	y	y	y
Chean et al.	y	y	y	y	y	n	y	y	y	y
Coleman, Murphy & Cheater	y	y	y	y	y	n	y	y	y	y
Coleman, Cheater & Murphy	y	y	y	y	y	n	y	y	y	y
Gonzalez et al.	y	y	y	y	y	n	y	y	y	y
Guassora and Baart	y	y	y	y	y	n	y	y	y	y
Guassora and Gannik	y	y	y	y	y	n	y	y	y	y
Guassora and Tulinius	y	y	y	y	y	n	y	y	y	Y

Halladay et al.	y	y	y	y	y	n	y	y	y	y
Heath et al	y	y	y	y	y	n	y	y	p	y
Holliday et al	y	y	y	y	y	n	y	y	y	y
Kerr, Watson & Tolson	y	y	y	y	y	n	y	y	y	y
Lin and Ward	y	y	y	y	y	n	y	y	y	y
Nowlin et al.	y	y	y	y	y	n	y	y	y	y
Pilnick and Coleman	y	y	y	y	y	n	y	y	y	y
Van Rossem et al.	y	y	y	y	y	n	y	y	y	y
Wilson et al.	y	y	y	y	p	n	y	y	y	y

[1]CASP comprises 10 questions: two screening questions about the aims of the research and appropriate use of a qualitative methodology, and eight questions covering research design, sampling strategy, data collection, researcher's reflexivity, ethical issues, data analysis, the findings, and the value of the research. Two authors (JS and EM) performed this assessment independently and then discussed the results within the research group until we reached agreement. Given the lack of consensus about the role and function of study quality assessment as part of systematic reviews, we did not exclude any study from the analysis based on our evaluation. However, Results are as reliable as they are based on the studies with high methodological quality. We therefore performed a secondary sensitivity analysis by excluding from the synthesis studies in the lowest quartile of methodological quality. The quality appraisal showed that the overall quality of the studies was high. Several papers failed to address the role of the researchers, that is, their own possible effects on the findings and/or interpretations (reflexivity item); others failed to report data collection and data analysis sufficiently.

Supplementary Table 3. CERQual of Review Findings

Summary of review findings	Studies contributing to the review findings	Methodological limitations	coherence	adequacy	relevance	Assessment of the confidence in the evidence	Explanation of CERQUal assessment
What lacks?							
Patients' lack of motivation	40	Minor methodological limitations (one study with minor methodological limitations (no reflexivity))	No or very minor concerns about coherence	Serious concerns about adequacy (only one study, offering thin data)	Moderate concerns about relevance (partial relevance as the study was from only one setting)	Low confidence	Minor concerns regarding methodological limitations and coherence, moderate concerns regarding relevance and serious concerns regarding adequacy
PCPs' lack of sincerity and adequacy	41, 42	Minor methodological limitations (both studies had minor methodological limitations (no reflexivity))	No or very minor concerns about coherence	Serious concerns about adequacy (only two studies, both offering thin data)	Moderate concerns about relevance (partial relevance as the studies were from only two settings)	Low confidence	Minor concerns regarding methodological limitations and coherence, moderate concerns regarding relevance and serious concerns regarding adequacy
Lack of support	43	Minor methodological limitations (one study with minor methodological limitations (unclear recruitment and sampling strategy))	No or very minor concerns about coherence	Serious concerns about adequacy (only one study, offering thin data)	Moderate concerns about relevance (partial relevance as the study was from only one setting)	Low confidence	Minor concerns regarding methodological limitations and coherence, moderate concerns regarding relevance and serious concerns regarding adequacy
Lack of time and of a common time frame	44, 45, 46	Minor methodological limitations (3 studies with minor methodological limitations (no reflexivity))	No or very minor concerns about coherence	Moderate concerns about adequacy (only 3 studies)	Minor concerns about relevance (3 studies with direct relevance)	High confidence	Minor concerns regarding methodological limitations, coherence and relevance, moderate concerns about adequacy

Lack of skills and training	43, 47, 48	Minor methodological limitations (2 studies with minor methodological limitations (no reflexivity) and one with moderate methodological limitations (unclear recruitment and sampling strategy, no reflexivity))	No or very minor concerns about coherence	Moderate concerns about adequacy (only 3 studies)	Minor concerns about relevance (3 studies with direct relevance)	Moderate confidence	Minor concerns regarding methodological, coherence and relevance, moderate concerns regarding adequacy
Some expectations but no request							
Patients' expectations about PCPs	42, 44, 46, 49, 50, 51, 52, 53, 54, 55	Minor methodological limitations (8 studies with minor methodological limitations (no reflexivity) and 2 with moderate methodological limitations (unclear research design, unclear statement of data collection, no reflexivity))	No or very minor concerns about coherence	No or very minor concerns about adequacy (10 studies that together offered moderately rich data)	Minor concerns about relevance (studies of lay patients who smoked across three continents and including a fairly wide range of patients who smoked)	High confidence	Minor concerns regarding methodological limitations, coherence, adequacy and relevance
PCPs roles and attitude	43, 44, 45, 46, 51, 56, 57	Minor methodological limitations (6 studies with minor methodological limitations (no reflexivity) and 1 with moderate methodological limitations (insufficiently rigorous data analysis))	No or very minor concerns about coherence	No or very minor Concerns about adequacy	Minor concerns about relevance (7 studies with direct relevance)	High confidence	Minor concerns regarding methodological limitations, coherence, adequacy and relevance
How to address the issue and induce patients' motivation?							

Timing and temporality							
The right moment	44, 45, 51, 54, 58, 59	Minor methodological limitations (6 studies with minor methodological limitations (no reflexivity))	No or very minor concerns about coherence	No or very minor Concerns about adequacy	Minor concerns about relevance (studies of lay patients who smoked and PCPs across three continents)	High confidence	Minor concerns regarding methodological limitations, coherence, adequacy and relevance
Familiarity, continuity and trust	41, 43, 45, 49, 52, 54, 55, 60	Moderate methodological limitations (6 studies with minor methodological limitations (no reflexivity) and two with moderate (unclear data collection and recruitment and sampling strategy))	No or very minor concerns about coherence	No or very minor Concerns about adequacy	Minor concerns about relevance (studies of lay patients who smoked and PCPs across three continents and including a fairly wide range of different participants)	Moderate confidence	Minor concerns regarding coherence, adequacy and relevance, moderate concerns regarding methodological limitations
Strategies and approaches							
Testing the water, small talk, or holistic method	43, 44, 45, 50, 57, 60, 61	Minor methodological limitations (4 studies with minor methodological limitations (no reflexivity) and 3 studies with moderate methodological limitations (1 with unclear data collection, 1 with unclear research design and 1 with unclear recruitment and sampling strategy))	No or very minor concerns about coherence	No or very minor Concerns about adequacy	Minor concerns about relevance (studies of lay patients who smoked and PCPs across two continents, but only one was in North America and 6 in Europe)	Moderate confidence	Minor concerns regarding coherence, adequacy and relevance, moderate concerns regarding methodological limitations
Rational strategy and tangible link	45, 52, 59, 61	Minor methodological limitations (4 studies with minor methodological limitations (no reflexivity))	No or very minor concerns about coherence	No or very minor Concerns about adequacy	Moderate concerns about relevance (4 studies in the same continent, with direct relevance)	Moderate confidence	Minor concerns regarding methodological limitations, coherence and adequacy, moderate concerns regarding relevance

A “collaborative strategy”	43, 44, 49, 50, 51, 54, 61	Minor methodological limitations (4 studies with minor methodological limitations (no reflexivity) and 3 studies with moderate methodological limitations (1 with unclear data collection, 1 with unclear research design and 1 with unclear recruitment and sampling strategy))	No or very minor concerns about coherence	No or very minor Concerns about adequacy	Minor concerns about relevance (7 studies across two continents, with direct relevance)	High confidence	Minor concerns regarding coherence, adequacy and relevance, moderate concerns regarding methodological limitations
A “confrontational strategy”	43, 47, 49, 61	Minor methodological limitations (2 studies with minor methodological limitations (no reflexivity) and 2 with moderate methodological limitations (1 with unclear recruitment and sampling strategy and 1 with unclear research design))	No or very minor concerns about coherence	No or very minor Concerns about adequacy	Minor concerns about relevance (4 studies across two continents, with direct relevance)	Moderate confidence	Minor concerns regarding coherence, adequacy and relevance, moderate concerns regarding methodological limitations
Patient-centred approach	44, 47, 50, 52, 57	Minor methodological limitations (3 studies with minor methodological limitations (no reflexivity) and 2 with moderate methodological limitations (1 with unclear research design and 1 with insufficiently rigorous data analysis))	No or very minor concerns about coherence	No or very minor Concerns about adequacy	Minor concerns about relevance (5 studies across two continents, with direct relevance)	Moderate confidence	Minor concerns regarding coherence, adequacy and relevance, moderate concerns regarding methodological limitations
Educational approach	47, 54	Minor methodological limitations (2 studies with minor methodological limitations (no reflexivity))	No or very minor concerns about coherence	Serious concerns about adequacy (only 2 studies)	Moderate concerns about relevance (partial relevance as the studies were from only two settings)	Low confidence	Minor concerns regarding methodological limitations, coherence and adequacy, moderate concerns regarding relevance
Using addiction model	44, 54	Minor methodological limitations	No or very minor	Serious concerns about	Moderate concerns about relevance (partial relevance as the studies	Low confidence	Minor concerns regarding methodological limitations, coherence and adequacy,

		(2 studies with minor methodological limitations (no reflexivity))	concerns about coherence	adequacy (only 2 studies)	were from only two settings)		moderate concerns regarding relevance
Positive and targeted messages	40, 54	Minor methodological limitations (2 studies with minor methodological limitations (no reflexivity))	No or very minor concerns about coherence	Serious concerns about adequacy (only 2 studies)	Moderate concerns about relevance (partial relevance as the studies were from only two settings)	Low confidence	Minor concerns regarding methodological limitations and coherence, moderate concerns regarding relevance and serious concerns regarding adequacy
Carbon monoxide monitoring	54	Minor methodological limitations (1 study with minor methodological limitations (no reflexivity))	No or very minor concerns about coherence	Serious concerns about adequacy (only 1 study)	Moderate concerns about relevance (partial relevance as the studies were from only one setting)	Low confidence	Minor concerns regarding methodological limitations and coherence, moderate concerns regarding relevance and serious concerns regarding adequacy

Supplementary Table 4. Quotations from Patients, PCPs and Authors of primary studies to illustrate each theme

	What lacks
Patients' lack of motivation	P: "'The enjoyment of cigarettes, at the moment it outweighs any constant health considerations.' (40)
PCPs' lack of sincerity and adequacy	PCP: "When you are outside an addiction, it is easy to help… Why would anyone do it? It is wrong and gross et cetera, but when you're in the middle of an addiction, it can sometimes be harder to get perspective to throw it down to someone else when you are doing it yourself."(42)
Lack of support	PCP: "I work with tobacco cessation on my own initiative but I feel lonely in the role." (43)<br>A[PCP]: "The organization and the other team members did not always emphasize tobacco cessation activities as important." (43)
Lack of time and of a common time frame	P: "I'd have liked a prescription that day, I would ...I definitely would have liked that 'cos I would definitely have made an effort to stop smoking ... I feel I'd have went ahead with that. At that time 'cos I was a bit scared with my blood pressure and that and I felt that if I'd had that prescription I'd had the patches or something then I'd have tried to stop there and then but now it's gone into three weeks." (49)
Lack of skills and training	PCP: "I have gaps in my knowledge about the effect of snuff in general." (43)<br>PCP: "I think both the theoretical as well as the practical knowledge should be included in the dental hygienist education." (43)
	Some expectations but no request
Patients' expectations about PCPs	No expectation<br>P: "That smoking cessation guidance was possible at our healthcare centre? I had no idea!? But then it is also the question if I want to talk to my doctor about this." (46)<br>Own responsibility<br>P: "That smoking cessation guidance was possible at our healthcare centre? I had no idea!? But then it is also the question if I want to talk to my doctor about this." (46)<br>Medical issue<br>P: "You don't walk in and they go 'You don't have athlete's foot do you? Because I have some cream here' ... So, like, if you go to them and you say, 'Doc, I guess I've got some athlete's foot' and then he'll go 'Okay, I got some cream here' but for them to just kind of like come out and presume" (44)<br>Routinely topic<br>P: 'That support should be offered as soon as the doctor knows you're a smoker' (44)<br>Non-judgmental approach<br>P: ''I think... Well, I don't know exactly where the limit is, but I think there's a subtle line somewhere, where the GP should take care not to feel superior to the patient, well it's difficult to explain...'' (51)<br>Active role from PCPs<br>P: "The doctor would have to present facts, either using photos or tell stories from his professional career that he used to have such patients and they gave up… or that those who didn't, for example, are already dead." (52)<br>P: "I wanted to know the options I could do ... to stop smoking" (49).<br>GPs best place<br>P: "The only person who is in the position to help me is that doctor because I trust him. I'm not going to let somebody help me get rid of my addiction

	that takes up 2 hours a day that I've been doing for over 30 years that I don't trust" (54)<br>P: "The best in my case would be if the GP asked me if I want to give up smoking, if I smoke…, then I could possibly ask: What is good and effective advice to quit, Doctor?" (52)
PCPs roles and attitude	Objective: plant a seed<br>PCP: "The cessation must be seen in the long term. Planting a thought is very worthwhile." (43)<br>Provide advice but not motivate<br>PCP: "I know we are going towards primary prevention. In addition, I think that is good, but we have to be careful not to treat everything and think that we can control everything. And, if I am honest, I rather put my energy into someone who is very ill, than into a smoker who does not want to quit."(46)<br>Waiting for an explicit request<br>PCP: "If you actually took the ones who actually walk through the door and said I want help to stop smoking ... then I think I'm just ... probably as good as anybody else." (45)<br>Not "if" but "how"<br>PCP: "If someone expresses an interest [in quitting smoking] ... then I'll say, you know, ''There's a bunch of stuff out there to help you. There's the patch, there's Zyban''. Like, I will usually list the options if they do seem interested'" (44)<br>A^{mixte}: "The present study addresses the practical implementation of advice from the perspective of a single consultation, thereby shifting the focus from 'if' to 'how' advice occurs." (45)
	How to address the issue and induce patients' motivation
Timing and temporality	The right moment<br>P: "That's also something that can really be a part of … making the asthma worse, that you put smoke down there, that makes the body react, you know." (45)<br>P: "I'm on statins for cholesterol, and I've been getting a lot of joint pain. . . . I said, 'Look, I am done with these statins.' . . . And she said, 'I absolutely can't take you off the statins because as a smoker your risk of a heart attack is like 34% more" (54)<br>P: "Building and maintaining trust was an integral goal of consultations […] The finding of the present study that smoking cessation advice by GPs could be perceived as a lack of recognition of difficult psychosocial circumstances and thereby put trust under strain concurs with the finding in a study of trust and smoking cessation advice in antenatal visits with midwives." (57)<br>P: "The only person who is in the position to help me is that doctor because I trust him. I'm not going to let somebody help me get rid of my addiction that takes up 2 hours a day that I've been doing for over 30 years that I don't trust" (54).<br>P: "And so it's sort of been like a slap in the face sort of thing, like, 'Oh wow, I didn't realise it was doing that much damage'. " (59)<br>PCP: "GP B: Yes well, I would like it if you had one made here as well to see the …how you … . I seem to remember that you ' re a smoker, aren ' t you? How much do you smoke?/ P: I think I ' ve reduced it to ten now./ GP B: Ten, yes. That ' s also something that can really be a part of …making the asthma worse, that you put smoke down there, that makes the body react, you know." (45)<br>A^{P}: "The GP may have a role in helping when the time is right" (58)<br>A^{PCPs} : "The concept of a 'teachable moment' is described as 'a particular set of circumstances which leads individuals to alter their health behaviour positively'". (59)<br>Familiarity, continuity and trust<br>PCP: "When I give patients advice . . . I advise them as if I had even more right to advise them. Because I've suffered first from tobacco, the expense, giving up smoking and taking it up again.We need to be able to differentiate whether we are working or we are in our private life" (41).<br>PCP: 'You have to be sensitive. There are some doctors that tend to make patients feel like they're somehow morally culpable for their smoking, like

	they're doing something that is a sin. You're a bad person because you smoke, you're dirty, and your breath smells, and all that sort of crap. I tell people, 'listen, I know what it's like to enjoy smoking. It's not like its hurting anyone else as long as you do it in the open,' but I tell them its bad for you, its hurting your body, that's why we need to talk about this'. (47).<br>A^PCP: "This imbalance reflects an ethical dilemma with a tension between the dental hygienist's wish to provide successful oral health care work and the need to respect the patient's autonomy. The conflict may result in the use of power instead of open-minded communication at the expense of a good relationship with the patient. The patients' decisions need to be respected according to the principle of autonomy, even if a good treatment outcome is not always achieved." (43)
Stratégies and approaches	PCP: 'I try to use scare tactics to a certain extent. I usually talk about how it's gonna kill you.. You're gonna have a shorter life expectancy if you continue to smoke. Occasionally, if you have the time . . . I always say, "have you ever seen anyone on oxygen?" Most people have, [so] I say you're going to be just like that person if you continue to smoke' (47).<br>Patient centred approach<br>P: "If only there was such a tailored approach… If only I felt that this is only for me…maybe I would consider it [giving up smoking]" (52)<br>P: "[The GP] should treat everyone individually, be able to relate to the patient's disorders because we wouldn't care about something that is routine. If we keep on always hearing the same things at the doctor's, we'll stop listening. It depends on the way the doctor approaches the patient, his ability to influence the patient…" (52)<br>P: « Society needs more connection with people . . . I don't want to check yes or no. I want you to ask me my true need and to give me something to help it and then I want you to follow up. . . . Have one of your nurses call "Hey, how's that working or how are you feeling?" 'Cause there's many times I've gone home . . . on some medication and it was not right » (54)<br>PCP: 'I suggest things like why don't you try to cut down to 1 or 2 cigarettes per week or once every 2 weeks and work your way down from there. Then, if they're not quite so keen on the idea of cutting down, I suggest nicotine patches or nicotine gum. Unfortunately a lot of the times that's not an option for our patients because they're expensive'. (47)<br>A^mixte: "In this way they [GPs] would openly negotiate whether smoking cessation should be part of the discussion" (51).<br>Educational approach<br>P: "A lot of times when doctors give you things, it ends up in the car seat and I never read it. If they give it to you when you walk in the examination room, you'd literally have something to read while you're sitting there" (54)<br>PCP: 'I always include that tobacco is a contributor to medical problems the patient has, for example if a patient has high blood pressure and they're also a smoker. I kind of evaluate their knowledge before I go into my spiel about smoking cessation. So I'll ask are you aware that for example tobacco exacerbates high blood pressure, or can contribute to hardening of the arteries in conjunction with your diabetes . . .' (47)<br>Using addiction model<br>P:. "It's just like drug addiction . . . [it] has to be a part of the disease model, and they have to accept it as such" (54)<br>Positive and targeted messaging<br>P: "Listen, just say the word, we have plenty of things for you to help and just let me know; this is what works" (54)<br>P: 'The more the media try to bully me into giving up, the more I feel, sod them1 I'm not going to do it.' (40)<br>Carbon monoxide monitoring<br>P: "It's that affirmation model. It's something that's giving you that positive feedback. . . . You met that goal . . . I'm proud of you" (54)

P= Patients; PCP=PCP; A^P= Authors (studies with patients); A^PCP= Authors (studies with PCPs); A^mixte = Authors (stuides with patients and PC

Supplementary Table 5: Algorithm Complete search for each database

Database (results)	Phrase
Pubmed (262)	(("tobacco" [MeSH] OR "Smoking Cessation"[Mesh] OR "Smoking Reduction"[Mesh] OR "Tobacco Use Cessation"[Mesh]) AND (“qualitative research”[Mesh] OR “Nursing Methodology Research”[Mesh] OR “Focus Groups”[Mesh] OR “observation”[Mesh] OR “qualitative research” OR “qualitative study” OR “qualitative method”) AND (“perception” OR “attitude” OR “feeling” OR “knowledge” OR “belief” OR “view” OR “perspective” OR “opinion” OR “experience” OR “image” OR "self concept" OR “barrier” OR “management” OR “organization*” OR "Attitude of Health Personnel"[Mesh] OR "Attitude to Health"[Mesh] OR "Knowledge"[Mesh] OR "Psychology"[Mesh] OR "Self Concept"[Mesh] OR "Health Services Administration"[Mesh]) AND (“smokers“ [MeSH Terms] OR "Physician-Patient Relations"[Mesh] OR "Physicians"[Mesh] OR "Health Personnel"[Mesh] Or “Health care providers” OR “health professionals” OR “physicians, primary care“ [MeSH Terms] OR “general practice“ [MeSH Terms] OR "Dentist-Patient Relations"[Mesh] OR "Patients"[Mesh] OR “client“ OR “Professional-Patient Relations” OR "Motivation"[Mesh] OR "Pregnant Women"[Mesh] OR "Nurses"[Mesh] OR "Licensed Practical Nurses"[Mesh] OR "Nurses, Public Health"[Mesh]))
PsycINFO (5710)	((DE "Smoking Cessation" OR DE "Tobacco Use Disorder" OR DE "Tobacco Smoking" OR "Smoking Reduction") AND (DE "qualitative research" OR DE "observation methods" OR DE "grounded theory" OR DE "content analysis" OR DE "interviews" OR DE "group discussion" OR DE "hermeneutics" OR DE "narratives" OR DE "self-report" OR "focus groups" OR "observation*" OR "observation method*" OR "grounded theory" OR "content analysis" OR "interview*" OR "group discussion" OR "hermeneutics" OR "narratives" OR "self-report" OR "self report" OR "analytical research" OR "discourse analysis" OR "thematic analysis" OR "speech analysis" OR "observational study" OR "constant comparative method" OR "qualitative" OR "thematic" OR "empirical" OR "descriptive") AND ("study" OR "studies" OR "research" OR "method*" OR "report*" OR "approach" OR "interview*") AND (DE "Nurses"OR DE "Public Health Service Nurses" OR DE "Physicians" OR DE "Health Personnel" OR DE "General Practitioners" OR DE "Primary Health Care" OR “Health care providers” OR “health professionals” OR "Patients" OR “client“ OR "Dentist-Patient Relations" OR “ Professional-Patient Relations” OR "smokers" OR DE "Motivation" OR DE "Behavioral Intention" OR DE "Pregnancy" OR DE "Perinatal Period" OR DE "Postnatal Period" OR DE "Prenatal Care"))

Cinahl (1973)	(((MH "Qualitative Studies+") OR (MH "Focus Groups") OR (MH "Interviews+") OR (MH "Narratives") OR (MH "Observational Methods+") OR (MH "Discourse Analysis") OR (MH "Thematic Analysis") OR (MH "Semantic Analysis") OR (MH "Field Studies") OR (MH "Audiorecording") OR (MH "Constant Comparative Method") OR (MH "Content Analysis") OR (MH "Field Notes") OR "qualitative research" OR "qualitative study" OR " qualitative method")) AND ((MM "Smoking Cessation+") OR (MH "Tobacco Use Cessation products+") OR "Tobacco Use Cessation" OR "Tobacco Use Disorder" OR "Tobacco")<br>AND ((MH "Nurses+)" OR (MH "Practical Nurses") OR (MH "Community Health Nursing+") OR (MH "Physicians+") OR (MH"Health personnel+") OR "health care providers" Or "Health professionals" OR (MH "Physicians, Family") OR (MH "Primary Health Care") OR (MH "Patients+") OR (MH "Professional-Client Relations+") OR "client" OR (MH "Dentist-Patient Relations") OR (MH "Professional-Patient Relations+") OR (MH "Nurse-Patient Relations") OR (MH "Physicians-Patient Relations") OR "smokers" OR (MH "Motivation+") OR (MH "Intention") OR (MH "Postnatal Period+") OR (MH "Pregnancy+")))
SSCI (2995)	(("qualitative" OR "thematic" OR "empirical" OR "descriptive" OR "analytical") AND ("study" OR "studies" OR "research" OR "method*" OR "report*" OR "approach" OR "interview*" OR "focus groups" OR "observation" OR "grounded theory" OR "content analysis" OR "interviews" OR "discourse analysis" OR "thematic analysis" OR "speech analysis" OR "observational study" OR "constant comparative method" OR "hermeneutics" OR "narratives" OR "self-report" OR "self report" OR "analytical research") AND ("Smoking Cessation" OR "Smoking Reduction" OR "Tobacco Use Cessation" OR "Tobacco Use Disorder" OR "Tobacco") AND ("Nurses" OR "Licensed Practical Nurses" OR "Physicians" OR "Health Personnel" Or "Health care providers" OR "health professionals" OR "General Practitioners" OR "Primary Health Care" OR "Patients" OR "client" OR "Dentist-Patient Relations" OR "General Practice" OR "Professional-Patient Relations" OR "smokers" OR "Motivation" OR "Behavioral Intention" OR "Pregnancy" OR "Perinatal Period" OR "Postnatal Period" OR "Prenatal Care"))

Supplementary figure 1 : Extraction Data Sheet

<table>
<tr><td colspan="2">Citation</td><td colspan="10" rowspan="2"></td></tr>
<tr><td>N.</td><td></td></tr>
<tr><td colspan="2">Journal Impact Factor</td><td colspan="10"></td></tr>
<tr><td colspan="12">Critical Appraisal Skills Program</td></tr>
<tr><td>Aims</td><td>Method</td><td>Research design</td><td>Sampling</td><td>Data collection</td><td>Reflexivity</td><td>Ethical issues</td><td>Data Analysis</td><td>Findings</td><td>Value of research</td></tr>
<tr><td></td><td></td><td></td><td></td><td></td><td></td><td></td><td></td><td></td><td></td></tr>
<tr><td colspan="12">Characteristics</td></tr>
<tr><td>Focus or aim of the study</td><td>Methodology</td><td>Participants</td><td>Data collection</td></tr>
<tr><td></td><td></td><td></td><td></td></tr>
<tr><td>1st order results (results)</td><td colspan="3"></td></tr>
<tr><td>2nd order results (discussion)</td><td colspan="3"></td></tr>
<tr><td>Notes</td><td colspan="3"></td></tr>
</table>
